# Fracture of first rib after sternotomy

**DOI:** 10.4103/0019-5049.79886

**Published:** 2011

**Authors:** Dheeraj Arora, Yatin Mehta

**Affiliations:** Institute of Anaesthesiology and Critical Care, Medanta-The Medicity, Gurgaon, India

Sir,

Rib fracture is a known complication following median sternotomy mainly attributed to excessive retraction.[1,2] The symptoms depend upon the location of the fracture and associated brachial plexus injury. Sometimes symptoms persist because of occult rib fracture requiring special investigations like bone scan.[3]

**Figure 1:**
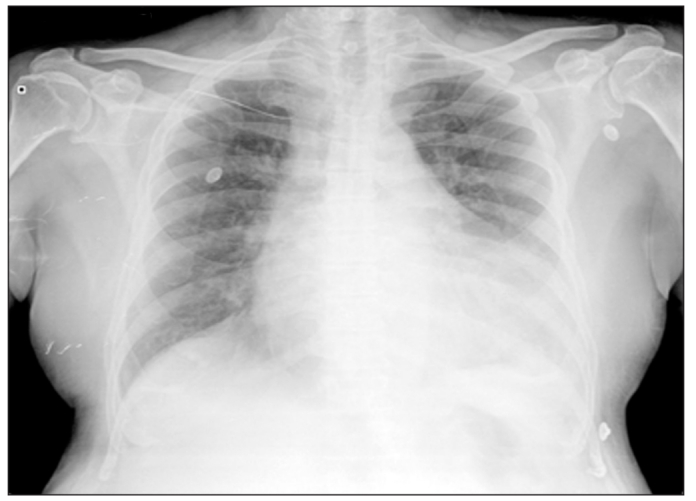
Preoperative chest radiograph

**Figure 2:**
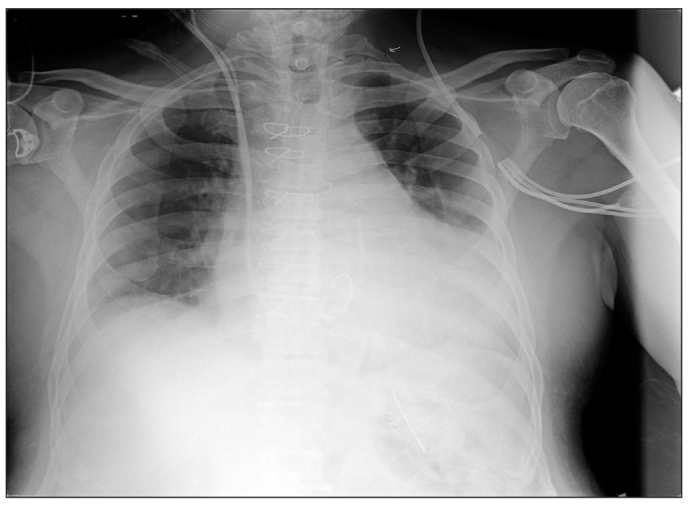
Postoperative chest radiograph showing fracture of left first rib

A 71-year-old gentleman with coronary artery and ischemic mitral regurgitation presented for coronary artery bypass grafting (CABG) and mitral valve repair. He was suffering from hypertension and chronic renal disease. His preoperative investigations revealed serum creatinine of 1.8 mg/dl and blood urea of 109 mg/dl. The rest of the investigations were within normal limits. Chest radiograph revealed cardiomegaly and congested lung fields [Figure 1]. Echocardiography showed global hypokinesia of the left ventricle with ejection fraction of 30% and moderate to severe mitral regurgitation with predicted pulmonary artery pressure (PAP) of 50 mmHg. He underwent CABG with three saphenous vein grafts and mitral valve repair with Carpentier Edwards classic ring. Total cardiopulmonary bypass (CPB) and aortic cross clamp time was 161 and 108 min respectively. Total surgical time was 350 min. Sternal retraction was done with double-blade retractor. Intraoperative period was uneventful. During the postoperative period, the patient was haemodynamically stable with minimal inotropic support. Postoperative portable chest radiograph revealed fracture of the left first rib [Figure 2]. Patient was electively ventilated for 16 h postoperatively in view of high PAP and combined surgical procedure. Patient did not have pain, numbness or parasthesia in the left upper limb. There was no evidence suggestive of associated brachial plexus injury.

Rib fracture is common after sternotomy and often goes unnoticed. The symptoms depend on location of the fracture. One-third of the fractures are reported each in the first and second rib and the remaining third throughout the thorax.[3] Occult rib fractures are often a major cause of non-incisional chest pain in patients who have undergone sternotomy.
